# Disproportionality Analysis of Renal Adverse Events Associated with a Combination of Immune Checkpoint Inhibitors and Acid-Suppressing Agents—A Pharmacovigilance Study Based on the FAERS Database

**DOI:** 10.3390/jcm14103581

**Published:** 2025-05-20

**Authors:** Jinmei Liu, Xu Chen, Cong Zhang, Huiping Hu, Shijun Li, Zhiwen Fu, Ruxu You

**Affiliations:** Department of Pharmacy, Union Hospital, Tongji Medical College, Huazhong University of Science and Technology, Wuhan 430022, China; liujinmeiu0817@163.com (J.L.); littlefourteen@126.com (X.C.); whxhzhc@163.com (C.Z.); huhuipingxh@163.com (H.H.); 04yaolishijun@163.com (S.L.)

**Keywords:** immune checkpoint inhibitors, nephrotoxicity, proton pump inhibitors, pharmacovigilance study, FAERS database

## Abstract

**Background/Objectives**: The nephrotoxicity of immune checkpoint inhibitors (ICIs) combined with proton pump inhibitors (PPIs) has been recognized but lacks a comprehensive analysis. We conducted an in-depth investigation of renal adverse events (rAEs) associated with ICIs and different acid-suppressing agents (ASAs)—including PPIs, histamine-2 receptor antagonists (H_2_RAs), and potassium-competitive acid blockers (P-CABs)—using real-world data from the FDA’s Adverse Event Reporting System (FAERS). **Methods**: We analyzed rAE reports from the FAERS database covering Q1 2004 to Q1 2023. Disproportionality analysis was conducted to identify rAEs associated with ICI or ASA monotherapy or combination therapy. Univariate logistic regression was employed to explore influencing factors. **Results**: No eligible rAE reports were retrieved for H_2_RAs and P-CABs. However, 6,775 reports in the ICI group, 54,055 reports in the PPI group, and 210 reports in the ICI–PPI combination therapy group were included in the final analysis. In PPI–ICI combination settings, tubulointerstitial nephritis had the highest reporting frequency and signal intensity; the overall risk of rAEs was significantly elevated compared to ICI or PPI monotherapy, with reporting odds ratios of 14. 65 (95% confidence interval [CI] 12.93–16.58) and 3.24 (95% CI 2.87–3.66), respectively; the median onset time was shortest at 21 days (interquartile range 5.5–135); and PD-1 monotherapy, omeprazole, and rabeprazole were associated with higher rAE risks. **Conclusions**: Our findings confirm that the combination of PPIs (but not other ASAs) with ICIs further increases the risk of various acute and chronic rAEs. Healthcare providers should exercise caution when managing patients on these therapies.

## 1. Introduction

Immune checkpoint inhibitors (ICIs) have transformed the treatment of solid and hematological tumors, becoming the first-line therapy [1]. Although their clinical efficacy is well established, their use is not without risk. Nephrotoxicity is a relatively uncommon immune-related adverse event (irAE) with an incidence range of 2–7% [2,3,4,5,6,7,8]. ICI-related renal injury can lead to serious consequences, including temporary or permanent discontinuation of ICIs or other concomitant anticancer therapies, the need for long-term corticosteroid therapy, and the requirement for ongoing renal replacement therapy [9].

Several risk factors have been identified in patients with ICI-associated AKI (acute kidney injury) or CKD (chronic kidney disease), including the concomitant use of medicines such as proton pump inhibitors (PPIs) [10,11], which have been described in multiple guidelines [12,13]. PPIs are a class of acid-suppressing agents (ASAs) mainly used to treat gastric acid-related diseases such as peptic ulcer disease and gastroesophageal reflux disease. As the most commonly used class of drugs worldwide, PPIs are also widely used in cancer patients, with a utilization rate of around 30% [14,15,16]. In a 10-year retrospective chart review, the combination of ICIs and PPIs was a significant independent predictor of AKI (Hazard Ratio [HR], 2.387; 95% confidence interval [CI], 1.328 ~ 4.291; *p* = 0.0036). The incidence of AKI was as high as 14.66% in the population of PPIs in combination with ICIs and 6.66% in ICIs alone [17]. In addition, in patients surviving ≥ 1 year after ICIs, PPIs were identified as an important risk factor for new-onset CKD, with a HR of 1.38 (95% CI, 1.10–1.73) [11]. Mechanistically, it has been hypothesized that PPIs may trigger a drug-specific T cell-mediated immune response. ICIs then reactivate these T cells, leading to the patient’s loss of tolerance to potentially nephrotoxic drugs [18]. However, the evidence of increased risks of PPIs for AKI and CKD progression among patients treated with ICIs is based on case reports or retrospective cohort studies and lacks comprehensive analysis. Furthermore, the other two kinds of ASAs, namely, histamine-2 receptor antagonists (H_2_RAs) and potassium-competitive acid blockers (P-CABs), are also considered to be both effective and safe, and there is no evidence of their harmful effect on renal function in the setting of ICI therapy.

Given the increasing use of ICIs in clinical practice and the potentially life-threatening outcomes of nephrotoxicity, it is critical to obtain accurate and comprehensive data on the clinical manifestations and prognosis of renal injury associated with the combination of ICIs and ASAs from a large population. The US Food and Drug Administration’s Adverse Event Reporting System (FAERS) serves as a valuable resource for identifying and monitoring safety signals of approved products [19]. By analyzing reports submitted to the FAERS, adverse events associated with specific therapies can be detected, enabling timely interventions to protect patient safety.

Herein, this study primarily aims to evaluate the association between the concomitant use of immune checkpoint inhibitors (ICIs) and acid-suppressing agents (ASAs) (including PPIs, H_2_RAs, misoprostol, and P-CABs) and the risk of renal adverse events (AEs) using the FAERS database while characterizing the clinical spectrum of associated nephrotoxicity. Additionally, we seek to identify risk factors, analyze the onset timing, and compare clinical outcomes of ICI–ASA nephrotoxic interactions. By addressing these objectives, this study aims to bridge critical knowledge gaps in the multifaceted renal risks of ICI–ASA combination therapy and provide actionable evidence for clinical monitoring and decision-making.

## 2. Materials and Methods

### 2.1. Study Design and Data Sources

This is a retrospective pharmacovigilance study comparing rAEs between three exposure groups: ICI monotherapy, ASA monotherapy (including PPIs, H_2_RAs, and P-CABs), and ICI + ASA combination therapy based on the FAERS database. In this study, ICIs (pembrolizumab, nivolumab, cemiplimab, durvalumab, avelumab, atezolizumab, tremelimumab, and ipilimumab), PPIs (omeprazole, lansoprazole, pantoprazole, rabeprazole, esomeprazole, and dexlansoprazole), H_2_RAs (ranitidine, cimetidine, famotidine, nizatidine, misoprostol, and roxatidine), and P-CABs (revaprazan, vonoprazan, tegoprazan, fexuprazan, and keverprazan) were used as keywords to obtain AE reports with these drugs as the primary suspect (PS) in the FAERS. Raw data from the first quarter of 2004 (Q1 2004) to the first quarter of 2023 (Q1 2023) were downloaded in ASCII format from the open FDA database in March 2024 and processed by the program to write to the SAS software (version 9.4). In the FAERS database, a reported AE case may have more than one report. Thus, the data cleaning procedure was conducted before data analysis. First, if the same CASEID (the number used to identify FAERS cases) was identified, the most recent FDA_DT (the date the case was received by the FDA) was kept. Second, if the same CASEID and FDA_DT were identified, the higher PRIMARYID (the unique number) was kept [19].

### 2.2. Identification of Target AE Reports

In the FAERS database, AEs are coded using a preferred term (PT) according to the Medical Dictionary for Regulatory Activities (MedDRA). PTs are unique descriptions of single medical concepts, which can be categorized into different System Organ Classes (SOCs) by etiology, site of origin, or cause. Standardized MedDRA Queries (SMQs) are groupings of PTs that relate to a defined medical condition or area of interest and were developed to optimize the detection and assessment of AE signals. We included a total of 151 rAE-related PTs for data analysis by combining SMQs and SOCs (Supplementary Materials, Table S1) to ensure that the analyzed PTs had no significant omissions and were real renal-related AEs from a clinical perspective.

### 2.3. Signal Mining

Disproportionality analysis is a widely used analytical method for pharmacovigilance studies [20]. This method can establish a statistical association for a given combination of drugs and AEs based on comparisons between the observed and expected numbers of reports. This study used the reporting odds ratio (ROR) and Bayesian confidence propagation neural network (BCPNN) to detect the PT signals of the abovementioned drugs (Supplementary Materials, Table S2). Only AE signals that met both ROR and BCPNN inclusion criteria were considered as positive signals for further analysis. Meanwhile, the ROR value can be used as an indicator to evaluate the risk level of adverse drug events. A higher ROR value indicates a stronger association between the target drug and the suspected AE. Due to the small number of AE reports detected in this study, the analysis was combined with statistical shrinkage transformation to protect against sharp associations and observe robust results. The shrink parameter was set to 0.5 [21,22].

We further analyzed the inclusion of positive PT signals in the PPI or ICI drug leaflet to better understand the nephrotoxicity after co-administration.

### 2.4. Descriptive Analysis

Clinical characteristics of rAE reports after screening were descriptively analyzed, including gender, age, country, diagnosis, outcome, year of reporting, drug, and time to onset of AEs. Time to AE onset was calculated by subtracting the date of event development (EVENT_DT) from the date of treatment start (START_DT) and was visualized using cumulative distribution curves to compare temporal patterns across exposure groups (ICI monotherapy, ASA monotherapy, and combination therapy). Reports with temporal inconsistencies (e.g., EVENT_DT preceding START_DT) or missing data were excluded.

### 2.5. Statistical Analysis and Reporting Guidelines

To assess the specificity of this association of the combination of ICIs and ASAs with an increased risk of rAEs, we first compared the RORs (using all other drug reports in the FAERS database as the reference) for rAEs among the ICI monotherapy, ASA monotherapy, and ICI + ASA combination therapy groups. Subsequently, using the ROR method, we compared the ICI–ASA combination therapy group against ICI monotherapy or ASA monotherapy groups, which were used as reference groups. This approach was crucial for understanding whether the observed association is specific to the ICI + ASA combination therapy group.

The Kruskal–Wallis test was used to estimate the difference in median time to the development of rAEs among the different groups. Univariate logistic regression was applied to explore factors that might influence the occurrence of rAEs in the ICI + PPI combination group. In all analyses, samples containing missing values were excluded; *p* < 0.05 was considered statistically significant, and all statistical tests were two-tailed. All statistical analyses and visualizations were performed using R software (version 4.3), IBM SPSS Statistics (version 27.0), and GraphPad Prism (version 10.0).

This study was conducted in accordance with the REporting of A Disproportionality Analysis for DrUg Safety Signal Detection Using Individual Case Safety Reports using PharmacoVigilance (READUS-PV) guidelines (Supplementary Materials, Table S3) [23].

## 3. Results

### 3.1. Scanning for rAEs in the FAERS Database

**Figure 1:**
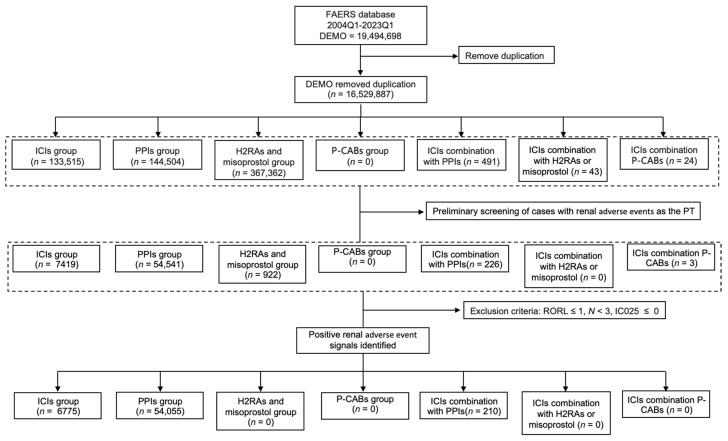
Flowchart showing the selection process of hepatitis adverse events for immune checkpoint inhibitors (ICIs) in the Food and Drug Administration’s Adverse Event Reporting System (FAERS). ICIs, immune checkpoint inhibitors; PPIs, proton pump inhibitors; P-CABs, potassium-competitive acid blockers; H_2_RAs, histamine-2 receptor antagonists; RORL, the lower limit of the 95% CI of the reporting odds ratio (ROR); and IC025, the lower limit of the 95% CI of the information component (IC).

From 2004 Q1 to 2023 Q1, a total of 16,529,887 unique AE reports were submitted to the FAERS database. We further searched for PTs of interest for rAEs (Supplementary Materials, Table S1) and screened for positive PT signals with RORL > 1, *N* ≥ 3, and IC025 > 0. Eventually, 6,775 reports in the ICI group, 54,055 reports in the PPI group, and 210 reports in the ICI–PPI combination group were included in the final analysis. No rAE reports for P-CABs or H_2_RAs were retrieved. The data processing procedure is detailed in Figure 1. 

A total of 16 positive PT signals were identified in ICI–PPI combination therapy and were compared to ICI and PPI monotherapy (Table 1). The top three reported rAEs among different treatment groups were as follows: AKI (*n* = 2121, 31.31%), renal impairment (*n* = 1089, 16.07%), and blood creatinine increased (*n* = 708, 10.45%) for ICIs alone; AKI (*n* = 22,727, 42.04%), tubulointerstitial nephritis (*n* = 5506, 10.19%), and renal impairment (*n* = 2622, 4.85%) for PPIs alone; and tubulointerstitial nephritis (*n* = 117, 55.71%), AKI (*n* = 104, 49.52%), and renal tubular acidosis (*n* = 22, 10.48%) in the case of ICIs combined with PPIs. However, when the ROR was used as a criterion, the top three AEs in each group changed significantly, as follows: immune-mediated nephritis (ROR = 113.44, 95% CI 80.64–159.57, *n* = 97), tubulointerstitial nephritis (ROR = 4.66, 95% CI 4.27–5.08, *n* = 523), and proteinuria (ROR = 3.91, 95% CI 3.54–4.32, *n* = 393) for ICIs alone; tubulointerstitial nephritis (ROR = 50.61, 95% CI 48.98–52.29, *n* = 5506), nephrosclerosis (ROR = 34.11, 95% CI 29.78–39.06, *n* = 287), and AKI (ROR = 16.63, 95% CI 16.40–16.87, *n* = 22,727) for PPIs alone; and tubulointerstitial nephritis (ROR = 115.74, 95% CI 95.87–139.73, *n* = 117), renal tubular acidosis (ROR = 40.60, 95% CI 26.58–62.01, *n* = 22), and glomerulonephritis (ROR = 23.88, 95% CI 13.81–41.29, *n* = 13) for combination therapy. The results showed that tubulointerstitial nephritis had a high intensity in all three groups.

It is worth noting that among the 16 positive signals, eight PTs—acute kidney injury, increased blood creatinine, glomerulonephritis, immune-mediated nephritis, nephrotic syndrome, proteinuria, renal impairment, and tubulointerstitial nephritis—were documented as adverse reactions in the prescribing information of ICIs (immune checkpoint inhibitors). Additionally, four PTs—acute kidney injury, increased blood creatinine, renal impairment, and tubulointerstitial nephritis—were listed in the prescribing information of PPIs (proton pump inhibitors) (Supplementary Materials, Table S4).

### 3.2. Descriptive Analysis of Cases with Renal AEs Under Different Treatments from the FAERS

**Figure 2:**
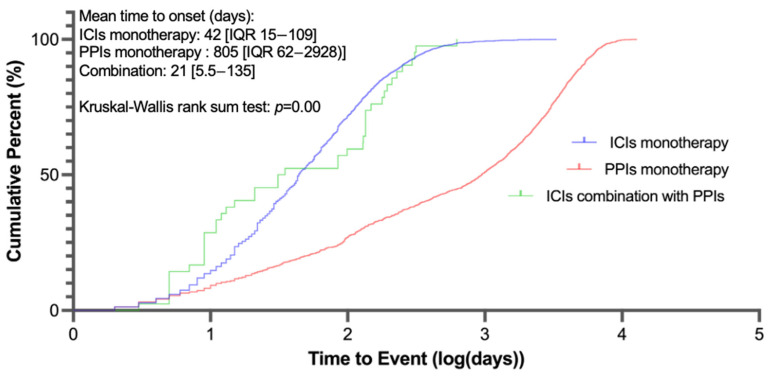
Cumulative distribution curves demonstrating the onset time of renal adverse events after different treatment strategies.

Baseline characteristics of patients (cases) with rAE reports are presented in Table 2. The ICI monotherapy group predominantly reported from Asia (38.6%), the Americas (32.13%), and Europe (27.45%), whereas over 80% of reports in the PPI monotherapy and combination therapy groups came from the Americas and Europe. In both the ICI monotherapy and combination therapy groups, around 50% of patients were male. The median age in the PPI group was comparable to the combination therapy group, and it was less than 70 years in the ICI monotherapy group. The most reported drugs were, in descending order, pembrolizumab (31.28%), nivolumab (27.99%), and nivolumab plus ipilimumab (18.27%) in the ICI group and esomeprazole (36.68%), lansoprazole (28.07%), and pantoprazole (19.4%) in the PPI monotherapy group. In the combination group, the most reported PPIs were omeprazole (48.1%) and pantoprazole (24.76%), and the most reported ICIs were nivolumab (42.38%) and pembrolizumab (29.05%). The shortest median onset time of 21 days (interquartile range [IQR] 5.5–135) was noted in combination therapy, with a significant difference confirmed by the Kruskal–Wallis rank sum test (Figure 2).

### 3.3. Signals of Disproportionate Reporting for ICI–PPI Combination Therapy Against Monotherapy

**Figure 3:**
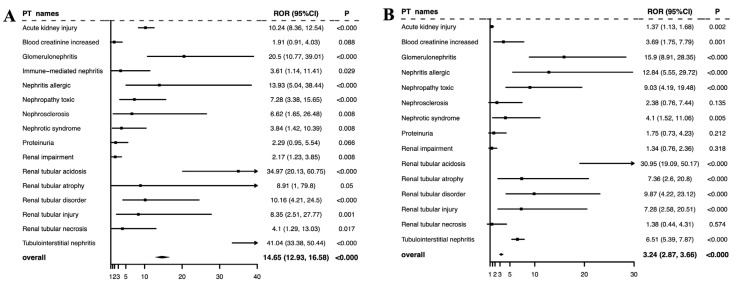
Signals of disproportionate reporting for renal AEs: (**A**) ICI–PPI combination therapy vs. ICI monotherapy and (**B**) ICI–PPI combination therapy vs. PPI monotherapy. Note: the arrows indicate that the upper limit of the confidence interval (CI) exceeds the predefined data range in the figure.

To verify whether the combination of ICIs and PPIs increased the risk of rAEs, we used the ROR method to compare ICI–PPI combination therapy against ICI monotherapy or PPI monotherapy groups, which were used as reference groups (Figure 3). The results showed that the overall risk of rAEs in the combination therapy group was significantly elevated compared to ICI or PPI monotherapy, with RORs of 14.65 (95% CI 12.93–16.58; *p* < 0.000) and 3.24 (95% CI 2.87–3.66; *p* < 0.000), respectively. These results suggest that the combination of ICIs and PPIs may increase the risk of rAEs compared to ICIs or PPIs alone.

### 3.4. Influencing Factors for rAEs in PPIs Combined with ICIs

**Figure 4:**
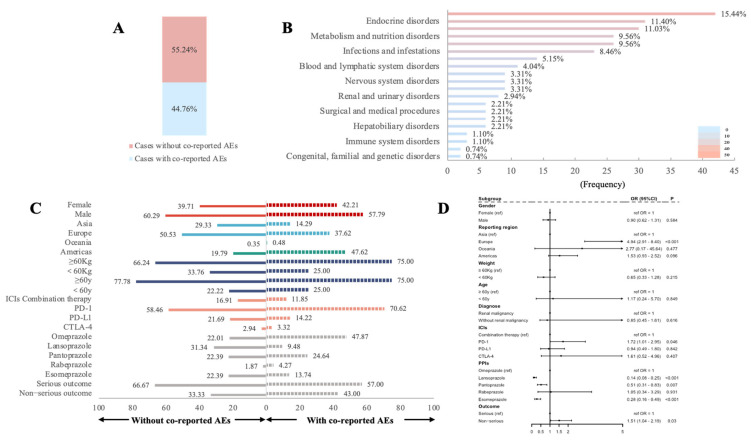
Factors influencing renal adverse events in ICI–PPI combination therapy. (**A**) Bar plot shows the proportion of cases with and without co-reported AEs in cases with renal AEs. (**B**) Bar plot shows the statistics of SOCs regarding PTs of co-reported adverse events. The percentage values labeled in the figure represent the proportion of cases with such adverse events out of the total renal AE cases with co-reported adverse reactions. (**C**) Bar plot shows the proportion of different subgroups of cases with or without co-reported AEs. (**D**) The forest plot shows the results of univariate logistic regression analysis regarding the factors influencing renal AEs. Note: OR: odds ratio. AE: adverse event. SOC: system organ class. The arrows in forest plot indicate that the upper limit of the confidence interval (CI) exceeds the predefined data range in the figure.

For the ICI–PPI combination therapy group, we further examined the factors that might influence the occurrence of rAEs. First, we analyzed co-reported AEs to explore whether they might be influential factors for rAEs. Of the 210 cases with rAEs, 55.76% were accompanied by other AEs (Figure 4A,B). Further, we conducted univariate logistic regression analysis (Figure 4C,D). No correlation was found between gender, age, diagnosis, or body weight and rAEs. When different ICIs were combined with PPIs, PD-1 monotherapy had the highest risk of rAEs, which was 0.72 times higher than that in the ICI combination group (OR = 1.72, 95% CI 1.01–2.95, *p* = 0.046). Among the different PPIs, lansoprazole (OR = 0.14, 95% CI 0.08–0.25, *p* < 0.001), pantoprazole (OR = 0.51, 95% CI 0.31–0.83, *p* = 0.007), and esomeprazole (OR = 0.28, 95% CI 0.16–0.49, *p* < 0.001) all had a lower risk of rAEs compared to omeprazole; rabeprazole (OR = 1.05, 95% CI 0.34–3.29, *p* = 0.931) had a comparable risk of rAEs with omeprazole (Figure 4D).

## 4. Discussion

The interaction between ICIs and other drugs and their potential impact on renal injury has received increasing attention from professionals [17,18,24]. To our knowledge, this is the first comprehensive pharmacovigilance study using real-world FAERS data to explore the reporting of rAEs associated with ICIs in combination with different ASAs. Notably, in our analysis, no positive rAE signal was obtained for H_2_RAs or P-CABs, either alone or in combination with ICIs. However, a significant association was observed between the concomitant use of ICIs and PPIs and an increased reporting of both acute and chronic rAEs.

According to the FAERS, rAEs were reported in 5.56% (7419/133,515) of cases involving ICIs over a 20-year period (2004–2023), whereas the proportion increased to 46.03% (226/491) in reports of patients treated with both ICIs and PPIs. In line with the findings of previous studies, our study suggests that patients receiving combination therapies may be at a higher risk of reported rAEs based on the ROR than those using ICIs or PPIs alone, with RORs of 14. 65 (95% CI 12.93–16.58, *p* < 0.000) and 3.24 (95% CI 2.87–3.66, *p* < 0.000), respectively.

Over time, various types of ICI-associated renal injury have been described, with AKI being the most commonly reported, usually attributed to acute interstitial nephritis [18,25,26]. Among the 16 positive PT signals in our analysis, AKI ranked among the top three AEs in terms of the number of reports, and tubulointerstitial nephritis showed a high signal intensity across groups. Two large retrospective studies have identified PPIs as an independent risk factor for ICI-related AKI, with a relative risk of 2.07 (95% CI 1.17 −3.66, *p* = 0.0124) and 2.85 (1.81 to 4.48), respectively [6,24]. However, in our study, the ROR for AKI in the combination group compared to the ICI monotherapy group was 10.24 (95% CI 8.36–12.54; *p* < 0.000), which is higher than the above results. However, this discrepancy may be partly explained by differences in the study design and reporting practices. For example, we found that 17.27% of the AE reports in the ICI monotherapy group were submitted by non-health providers, compared to only 1.9% in the combination group. Reports from non-health providers may be subject to misdiagnosis or underreporting, resulting in lower reported data than the actual occurrence. In addition, the limited sample size and short follow-up time of retrospective studies may have hindered the detection of rare AEs.

Existing studies on the epidemiology of nephrotoxicity have focused on AKIs, and the long-term renal effects of ICIs remain unclear. One study reported that 20% of patients treated with ICIs who survived at least one year developed new-onset CKD or a sustained decline in eGFR of >30% for more than 90 days [11]. In this study, we observed that in addition to AKI, the combination of PPIs and ICIs was associated with increased reporting of other CKD-associated rAEs, such as glomerulonephritis, immune-mediated nephritis, nephrotic syndrome, and so on (Figure 3). These findings highlight the need for further research into the long-term renal safety of combination therapy. However, it is important to emphasize that the combination of PPIs and ICIs did not appear to increase the risk of serious adverse outcomes (death or life-threatening) compared to ICIs alone, with a ratio of 15.72% vs. 30.75%. This is in line with prior studies, such as a retrospective study of 1615 patients that found that PPI co-administration increased the risk of AKI but not mortality in ICI-treated patients [24].

The latency period from ICI initiation to renal injury onset varies across studies, ranging from 1 to 10 months or longer [5,27,28]. In our study, the median time to rAE onset in the ICI monotherapy group was 42 days (IQR 15–109), consistent with the reported timeframe. More importantly, we observed for the first time that the combination of PPIs and ICIs was associated with a shorter median time to rAE onset (21 days, IQR 5.5–135) compared to PPIs (805 days, IQR 62–2928) or ICIs (42 days, IQR 15–109) alone. The results suggest that the combination of PPIs and ICIs not only increases the risk of kidney injury but may also significantly shorten the time to occurrence.

Previous studies have indicated that older age and the male gender are linked to a higher risk of ICI-related AKI [8,17,29]. In our analysis, no significant associations were found between gender, age, diagnosis, or body weight and rAEs in the combination of PPIs and ICIs. We did, however, observe that more rAEs were reported in Europe than in Asia (OR 4.94, 95%CI 2.91–8.40, *p* < 0.001), suggesting possible regional or racial differences. Current research on ethnic differences in renal injury from PPIs or ICIs is limited, with one study indicating that Asians may have a higher risk of AKI compared to White patients (HR 4.182, 95% CI 1.090 −16.043, *p* = 0.0370) [17]. However, in this study, only 12 Asians were included, representing 0.72% of the total population. Thus, the available results suggest racial differences in renal injury risk from PPIs or ICIs but are insufficient to define specific risks across races. Comprehensive global studies are needed to explore these disparities.

A systematic review reported that the presence of extrarenal irAEs, e.g., colitis, hypophysitis, rash, and pneumonitis, was strongly suggested as an important risk factor for AKI (pooled OR 2.34, 95% CI 1.53–3.59, *p* < 0.001). In our study, 44.76% of ICI-associated rAEs were accompanied by other concomitant AEs. Extrarenal irAEs as a risk factor for renal injury may be associated with a multisystemic response induced by inflammatory factors, but this hypothesis has not been confirmed [8].

When different ICIs were combined with PPIs, we found that PD-1 monotherapy combined with PPIs was associated with the highest risk of rAEs (OR 1.72, 95%CI 1.01–2.95, *p* = 0.046), contrasting with the common belief that combinations of anti-CTLA-4 and anti-PD-1 or anti-PD-L1 pose the greatest AKI risk [5,8,17]. This discrepancy potentially may have been influenced by the small sample size, the lack of cumulative dose, and differences in exposure periods in our analysis [30]. Similarly, the higher risk of renal injury for omeprazole and rabeprazole compared to other PPIs may be attributable to these limitations [31,32]. Bin Wu et al. found that dexlansoprazole was more strongly associated with CKD (ROR = 34.94, 95% CI 30.89–39.53) and AKI (ROR = 8.18, 95% CI 7.04–9.51) than the other PPIs, while omeprazole showed the weakest association (ROR = 1.27, 95% CI 7.04–9.51) with CKD [33]. In our study, omeprazole accounted for 48.1% of PPI use in the combination group, but there were no cases involving dexlansoprazole. It is also possible that the risk profile of individual PPIs changes when used with ICIs due to potential synergistic effects, as suggested by KATO et al., who reported a higher risk of nephritis with omeprazole combined with nivolumab or ipilimumab [34].

In addition to PPIs, H_2_RAs and P-CABs are also commonly used ASAs [35]. In contrast to PPIs, no positive rAE signals associated with H_2_RAs or P-CABs were mined in this study. A comparative study showed that PPIs significantly increased the risk of CKD by 1.3-fold compared to H_2_RAs, which were not significantly associated with CKD [36]. It has been suggested that H_2_RAs might offer a nephroprotection effect by decreasing plasma histamine levels and inflammatory responses [37]. Regarding P-CABs, no rAEs have been reported [38]. A Japanese pharmacovigilance study indicated that all PPIs, except for vonoprazan (a kind of P-CAB), increased the risk of nephritis when combined with ICIs. Given these findings, if PPI-induced nephrotoxicity is suspected, switching to another PPI may not be advisable due to the potential for recurrent nephrotoxicity. Instead, when necessary, H_2_RAs or P-CABs may be considered as alternatives [18].

Despite the strengths of this study, we acknowledge several limitations. First, similar to other spontaneous reporting systems, the FAERS database, which is accessible to health professionals and the general public globally, is subject to inherent selection and reporting biases, including underreporting and notoriety bias. Additionally, the number of AEs reported is influenced by the frequency of prescription and use of the respective drugs. Consequently, we were unable to establish a causal relationship between the target drug and the target PT or to calculate the incidence of rAEs. Second, there may be disproportionate reporting between different regions and populations, which could introduce geographic or ethnic bias and limit the generalizability of our findings. Third, there is a wide variety of medications that may adversely affect kidney function (including chemotherapeutic agents, targeted agents, nonsteroidal anti-inflammatory drugs, antibiotics, etc.). It was challenging to identify and adjust for all potential nephrotoxic co-medications based on the available data. Moreover, the database lacks detailed clinical information, including diagnostic confirmation, comorbidities, laboratory data, and concomitant medications, which limits our ability to control for confounding factors and may lead to biased or false-positive associations. Finally, some reports lacked key information, such as the onset time of rAEs, age, etc. Our analyses were therefore limited to cases with complete data, potentially affecting the accuracy and representativeness of our findings.

## 5. Conclusions

This study, leveraging real-world data from the FAERS database, identifies a strong association between the combination of PPIs and ICIs and increased reporting of renal adverse events, as well as a shorter time to onset. Despite the current limited evidence, this study underscores the necessity for enhanced monitoring and proactive management in clinical practice as ICIs gain wider clinical acceptance. However, due to the potential for confounding by indication and other limitations inherent to the spontaneous reporting of data, these findings should be interpreted with caution. Future prospective studies are warranted to better understand the causal relationships and underlying mechanisms.

## Figures and Tables

**Table 1:** Sixteen positive renal adverse event-associated PTs identified in the combination of ICIs and PPIs versus those reported in the monotherapy strategies in the FAERS from 2004 Q1 to 2023 Q1.

PT Names	rAEs Reported in Full Database	ICI Monotherapy			PPI Monotherapy			ICIs Combined with PPIs		
		rAEs Reported	ROR (95% CI)	IC025	rAEs Reported	ROR (95% CI)	IC025	rAEs Reported	ROR (95% CI)	IC025
Acute kidney injury	160,761	2121	1.86 (1.79, 1.95)	0.81	22,727	16.63 (16.40, 16.87)	3.77	104	18.22 (14.94, 22.22)	3.91
Increased blood creatinine	55,109	708	1.81 (1.68, 1.95)	0.72	455	0.81 (0.73, 0.88)	−0.46	7	3.13 (1.49, 6.57)	0.67
Glomerulonephritis	1917	33	2.38 (1.69, 3.36)	0.71	104	5.44 (4.46, 6.62)	2.09	13	23.88 (13.81, 41.29)	6.73
Immune-mediated nephritis	147	97	113.44 (80.64, 159.57)	6.20	0	N/A	N/A	3	6.93 (2.21, 21.79)	7.36
Nephritis allergic	304	8	3.23 (1.60, 6.53)	0.76	25	7.54 (5.01, 11.35)	2.37	7	14.70 (6.94, 31.15)	8.18
Nephropathy toxic	8183	115	1.97 (1.64, 2.37)	0.67	98	1.17 (0.96, 1.43)	−0.10	7	9.60 (4.57, 20.16)	3.43
Nephrosclerosis	1055	6	0.81 (1.80, 0.36)	−1.63	287	34.11 (29.78, 39.06)	4.54	3	6.53 (2.10, 20.29)	4.51
Nephrotic syndrome	5491	139	3.58 (3.02, 4.24)	1.55	177	3.21 (2.76, 3.72)	1.41	4	6.53 (2.45, 17.42)	2.80
Proteinuria	14,321	393	3.91 (3.54, 4.32)	1.78	781	5.57 (5.19, 5.99)	2.30	5	5.53 (2.3, 13.32)	1.91
Renal impairment	66,302	1089	2.32 (2.19, 2.47)	1.10	2622	4 (3.85, 4.16)	1.89	12	4.50 (2.55, 7.94)	1.47
Renal tubular acidosis	1609	30	2.57 (1.79, 3.69)	0.80	68	4.17 (3.27, 5.32)	1.66	22	40.60 (26.58, 62.01)	7.95
Renal tubular atrophy	720	1	0.26 (0.04, 1.88)	−5.56	33	4.41 (3.11, 6.25)	1.61	4	8.58 (3.21, 22.94)	5.73
Renal tubular disorder	2487	29	1.62 (1.13, 2.34)	0.11	47	1.85 (1.38, 2.47)	0.42	6	11.10 (4.98, 24.77)	4.82
Renal tubular injury	570	8	1.87 (0.93, 3.76)	−0.15	35	5.89 (4.18, 8.29)	2.04	4	8.66 (3.24, 23.19)	6.07
Renal tubular necrosis	7893	73	1.3 (1.03, 1.63)	−0.01	600	7.93 (7.29, 8.61)	2.76	3	4.53 (1.46, 14.08)	1.61
Tubulointerstitial nephritis	16,103	523	4.66 (4.27, 5.08)	2.04	5506	50.61 (48.98, 52.29)	5.02	117	115.74 (95.87, 139.73)	7.42

Data are *n* unless otherwise stated. The IC025 value (>0) and the lower limit of ROR (>1) are the traditional thresholds used in statistical signal detection with the FAERS. FAERS, the FDA’s Adverse Event Reporting System; ICIs, immune checkpoint inhibitors; PPIs, proton pump inhibitors; IC, information component; ROR, reporting odds ratio; 95% CI, 95% confidence interval; IC025, the lower end of the 95% credibility interval for the IC; and N/A, not applicable.

**Table 2:** Clinical characteristics of patients with renal AEs under different treatments from the FAERS.

Clinical Characteristics	ICI Monotherapy (*n* = 6775)	PPI Monotherapy (*n* = 54,055)	ICIs in Combination with PPIs (*n* = 210)
Reporting region			
Americas	2177 (32.13)	51,052 (94.44)	101 (48.1)
Oceania	103 (1.52)	120 (0.22)	1 (0.48)
Africa	15 (0.22)	8 (0.01)	0 (0)
Europe	1860 (27.45)	2415 (4.47)	77 (36.67)
Asia	2615 (38.6)	296 (0.55)	30 (14.29)
Missing	5 (0.07)	164 (0.3)	1 (0.48)
Reporters			
Healthcare professionals	5577 (82.32)	7110 (13.15)	204 (97.14)
Non-healthcare professionals	1170 (17.27)	28,700 (53.09)	4 (1.9)
Missing	28 (0.41)	18,245 (33.75)	2 (0.95)
Reporting year			
2004–2008	0 (0)	343 (0.63)	0 (0)
2009–2013	50 (0.74)	823 (1.52)	0 (0)
2014–2018	1830 (27.01)	13,349 (24.7)	32 (15.24)
2019–2023 Q1	4895 (72.25)	39,540 (73.15)	178 (84.76)
Gender			
Male	4245 (62.66)	13,313 (24.63)	115 (54.76)
Female	2052 (30.29)	18,291 (33.84)	82 (39.05)
Missing	478 (7.06)	22,451 (41.53)	13 (6.19)
Age at onset, years	70 (61−75); *n* = 737	61 (54−68); *n* = 527	60 (59.5−62); *n* = 16
Diagnosis of renal malignancy	114 (1.68)	11 (0.02)	19 (19.05)
Drugs			
ICIs	5510 (81.33)	N/A	186 (88.57)
Nivolumab	1896 (27.99)	N/A	89 (42.38)
Pembrolizumab	2119 (31.28)	N/A	61 (29.05)
Cemiplimab	70 (1.03)	N/A	0 (0)
Atezolizumab	961 (14.18)	N/A	18 (8.57)
Avelumab	74 (1.09)	N/A	2 (0.95)
Durvalumab	142 (2.1)	N/A	9 (4.29)
Ipilimumab	247 (3.65)	N/A	7 (3.33)
Tremelimumab	1 (0.01)	N/A	0 (0)
Combination therapy	1265 (18.67)	N/A	24 (11.43)
Nivolumab plus ipilimumab	1238 (18.27)	N/A	21 (10)
Pembrolizumab plus ipilimumab	15 (0.22)	N/A	3 (1.43)
Tremelimumab plus durvalumab	10 (0.15)	N/A	0 (0)
Atezolizumab plus Ipilimumab	2 (0.03)	N/A	0 (0)
PPIs			
Omeprazole	N/A	4875 (9.02)	101 (48.1)
Lansoprazole	N/A	15,174 (28.07)	21 (10)
Pantoprazole	N/A	10,486 (19.4)	52 (24.76)
Rabeprazole	N/A	867 (1.6)	9 (4.29)
Esomeprazole	N/A	19,828 (36.68)	29 (13.81)
Dexlansoprazole	N/A	2825 (5.23)	0 (0)
Time to rAE onset, days	42(15−109); *n* = 3033	805 (62−2928); *n* = 3261	21 (5.5−135); *n* = 50
Serious outcome			
Death	1317 (19.44)	6000 (11.1)	9 (4.29)
Life-threatening	766 (11.31)	632 (1.17)	24 (11.43)
Hospitalization	4147 (61.21)	6390 (11.82)	115 (54.76)
Disability	211 (3.11)	789 (1.46)	5 (2.38)
Others	5559 (82.05)	51,215 (94.75)	163 (77.62)
Missing	178 (2.63)	1012 (1.87)	1 (0.48)

Data are *n* (%) or median (IQR; range); ICIs, immune checkpoint inhibitors; PPIs, proton pump inhibitors; rAEs, renal adverse events; and N/A, not applicable.

## Data Availability

The data that support the findings of this study are available from the corresponding author (R.Y.) upon reasonable request.

## Supplementary Materials

The following supporting information can be downloaded at: https://www.mdpi.com/article/10.3390/jcm14103581/s1, Table S1: Renal adverse events associated preferred terms included in this study; Table S2: Formulas and signal inclusion criteria for ROR and BCPNN methods; Table S3: The READUS-PV checklist; Table S4: Inclusion of the 16 positive renal adverse events associated PTs identified in the combination of ICIs and PPIs in leaflet.

## Abbreviations

The following abbreviations are used in this manuscript:

ICIs	immune checkpoint inhibitors
PPIs	proton pump inhibitors
rAEs	renal adverse events
ASAs	acid-suppressing agents
H_2_RAs	histamine-2 receptor antagonists
P-CABs	potassium-competitive acid blockers
FAERS	the FDA’s Adverse Event Reporting System
HR	Hazard Ratio
CI	confidence interval
PS	primary suspect
PT	preferred term
SOC	System organ class
ROR	reporting odds ratio
AKI	acute kidney injury
CKD	chronic kidney disease
IQR	interquartile range
