# Supplementary material for: Disproportionality Analysis of Renal Adverse Events Associated with a Combination of Immune Checkpoint Inhibitors and Acid-Suppressing Agents—A Pharmacovigilance Study Based on the FAERS Database
Source: J Clin Med. 2025 May 20;14(10):3581. doi: 10.3390/jcm14103581 (PMC12112296; doi:10.3390/jcm14103581)
Supplement: Supplementary file 1 [file jcm-14-03581-s001.zip › jcm-3595971-supplementary.pdf]

Supplementary Material

Supplementary table 1 Renal adverse events associated preferred terms included in this study.

SMQ	SOC	PT name
Accidents and injuries	Renal and urinary disorders	Renal injury
Acute renal failure	Investigations	Renal function test abnormal<br>Blood urea nitrogen/creatinine ratio increased<br>Creatinine urine abnormal<br>Creatinine urine decreased<br>Kidney injury molecule-1
	Renal and urinary disorders	Acute kidney injury<br>Anuria<br>Nephritis<br>Oliguria<br>Prerenal failure<br>Renal impairment<br>Subacute kidney injury
	Surgical and medical procedures	Continuous haemodiafiltration
Acute renal failure/Chronic kidney disease	General disorders and administration site conditions	Oedema due to renal disease
	Investigations	Blood creatinine abnormal<br>Blood creatinine increased<br>Creatinine renal clearance abnormal<br>Creatinine renal clearance decreased<br>Glomerular filtration rate abnormal<br>Glomerular filtration rate decreased<br>Urine output decreased
	Metabolism and nutrition disorders	Hypercreatininaemia
	Renal and urinary disorders	Nephritic syndrome<br>Renal failure
	Surgical and medical procedures	Dialysis<br>Haemodialysis<br>Haemofiltration<br>Peritoneal dialysis<br>Renal transplant
Acute renal failure/Chronic kidney	Investigations	Protein urine present
	Renal and urinary	Azotaemia

disease/Tubulointerstitial diseases	disorders	Nephropathy toxic
		Proteinuria
		Tubulointerstitial nephritis
		Albuminuria
		Renal tubular disorder
		Renal tubular dysfunction
		Renal tubular injury
		Renal tubular necrosis
		Crystal nephropathy
Angioedema	Renal and urinary disorders	Perinephric oedema
Chronic kidney disease	Blood and lymphatic system disorders	Nephrogenic anaemia
	Cardiac disorders	Pericarditis uraemic
	Gastrointestinal disorders	Uraemic gastropathy
	Hepatobiliary disorders	Hepatorenal failure
	Immune system disorders	Dialysis amyloidosis
	Injury, poisoning and procedural complications	Dialysis related complication
		Haemodialysis complication
		Peritoneal dialysis complication
	Investigations	Biopsy kidney abnormal
		Urine albumin/creatinine ratio increased
		Urine protein/creatinine ratio increased
		Ultrasound kidney abnormal
	Musculoskeletal and connective tissue disorders	Chronic kidney disease-mineral and bone disorder
		Uraemic myopathy
	Nervous system disorders	Uraemic encephalopathy
	Renal and urinary disorders	Autoimmune nephritis
		Chronic kidney disease
		End stage renal disease
		Focal segmental glomerulosclerosis
		Glomerulonephritis
		Glomerulonephritis chronic
		Glomerulonephritis membranoproliferative
		Glomerulonephritis membranous
		Glomerulonephritis minimal lesion
		Glomerulonephritis proliferative
		Glomerulonephritis rapidly progressive
		Glomerulonephropathy
		Glomerulosclerosis
		Goodpasture's syndrome

		Hypertensive nephropathy<br>IgA nephropathy<br>IgM nephropathy<br>Immune-mediated nephritis<br>Immune-mediated renal disorder<br>Intercapillary glomerulosclerosis<br>Lupus nephritis<br>Mesangioproliferative glomerulonephritis<br>Nephropathy<br>Nephrosclerosis<br>Nephrotic syndrome<br>Obstructive nephropathy<br>Renal amyloidosis<br>Acquired cystic kidney disease<br>C3 glomerulopathy<br>Fibrillary glomerulonephritis<br>Ischaemic nephropathy<br>Reflux nephropathy
	Skin and subcutaneous tissue disorders	Uraemic pruritus
	Surgical and medical procedures	Dialysis device insertion
	Vascular disorders	Vascular calcification
Chronic kidney disease/Tubulointerstitial diseases	Investigations	Albumin urine present<br>Urinary casts present
	Renal and urinary disorders	Kidney fibrosis<br>Renal atrophy<br>Renal tubular atrophy<br>Kidney small<br>Microalbuminuria<br>Renal papillary necrosis
Drug reaction with eosinophilia and systemic symptoms syndrome	Renal and urinary disorders	Glomerulonephritis acute<br>Renal disorder
Embolic and thrombotic events	Renal and urinary disorders	Renal embolism<br>Renal vascular thrombosis<br>Renal artery thrombosis<br>Renal artery occlusion
Haemodynamic oedema, effusions and fluid overload	Renal and urinary disorders	Oedematous kidney<br>Perinephric collection
Haemorrhages	Renal and urinary disorders	Subcapsular renal haematoma<br>Renal cyst haemorrhage<br>Renal haematoma

Hypersensitivity	Renal and urinary disorders	Henoch-Schonlein purpura nephritis
Hypertension	Renal and urinary disorders	Renal hypertension
Immune-mediated/autoimmune disorders	Renal and urinary disorders	Pulmonary renal syndrome<br>Anti-glomerular basement membrane disease
N/A	Renal and urinary disorders	Single functional kidney<br>Renal hypertrophy<br>Nephrocalcinosis<br>Renal salt-wasting syndrome<br>Renal cyst ruptured<br>Renal pain<br>Nephroptosis<br>Pyelocaliectasis<br>Kidney perforation<br>Renal cyst<br>Renal lipomatosis<br>Kidney enlargement<br>Renal mass<br>Haematinuria<br>Renal colic
Renovascular disorders	Renal and urinary disorders	Renal necrosis<br>Renal cortical necrosis<br>Renal ischaemia<br>Glomerular vascular disorder<br>Renal vessel disorder<br>Nephroangiosclerosis<br>Renal arteriosclerosis<br>Renal artery dissection<br>Renal aneurysm<br>Renal artery stenosis<br>Renal artery arteriosclerosis<br>Pelvi-ureteric obstruction
Tubulointerstitial diseases	Investigations	Beta-N-acetyl-D-glucosaminidase increased
	Renal and urinary disorders	Cylindruria<br>Fanconi syndrome acquired<br>Nephritis allergic<br>Nephrogenic diabetes insipidus<br>Polyuria<br>Renal tubular acidosis<br>Renal glycosuria

	Tubulointerstitial nephritis and uveitis syndrome

N/A: No relevant records in the target drug data retrieved from the faers database or in the SMQ. SMQ, standardized MedDRA query; SOC, system organ classification.

Supplementary table 2 Formulas and signal inclusion criteria for ROR and BCPNN methods

Algorithms	Equation	Criteria
ROR	$ROR=(a+0.5)/(bc/d+0.5)$ 95% $CI=e^{\ln(ROR)\pm1.96(1/a+1/b+1/c+1/d)^{\wedge}0.5}$	$a \geq 3$， lower limit of 95% CI ＞1
BCPNN	$IC=Log_2(a+0.5)/[(a+b)(a+c)/(a+b+c+d)+0.5]$<br>$IC025=IC-3.3(a+0.5)^{-1/2}-2(a+0.5)^{-3/2}$<br>$IC075=IC+2.4(a+0.5)^{-1/2}-0.5(a+0.5)^{-3/2}$	IC025 > 0

Notes: Equation: a, number of reports containing target adverse events of target drugs; b, number of reports containing other adverse events of the target drug; c, number of reports containing the target adverse events of other drugs; d, number of reports containing other adverse events of other drugs.

Abbreviations: ROR, reporting odds ratios; BCPNN, Bayesian confidence propagation neural network; 95% CI, 95% confidence interval; IC, information component; IC025, the lower limit of 95% CI of the IC.

Supplementary table 3 The READUS-PV checklist

Section and topic	Item #	Checklist item	Location where item is reported
**Title**			
	1a	*If disproportionality analyses are a prominent component of the published study, the study should be identified as a "disproportionality analysis". The type of data and name of the database(s) should be specified.*	P1
	1b	*Report the name of adverse event(s) and/or drug(s) under study, when applicable.*	P1
**Introduction**			
Background	*2a*	*Describe the drug(s) and its utilization, the nature of the adverse event(s) under study and its frequency, and the existing knowledge on the drug-event combination.*	P2
	2b	*Specify the rationale for performing the analysis, e.g., as part of routine pharmacovigilance, to investigate an overall safety profile, or to assess a pre-specified*	P2

		hypothesis.	
	2c	*Explain why ICSR databases and disproportionality analysis are suitable to fill the knowledge gap.*	P2
Objectives	*3*	*State specific objectives, identifying the adverse event(s), the drug(s), and the reference group, including any pre-specified hypothesis, if applicable.*	P2
**Methods**			
Study design	*4a*	*Identify the study (i.e., "disproportionality analysis") and the type of data used (e.g., "individual case safety reports").*	P3
	4b	*Provide an outline of the entire study design, including primary and sensitivity analyses performed, and other designs such as case-by-case analysis or literature review.*	P3-4
Data description, access, and pre-processing	*5a*	*Specify the name of the database(s), the database(s) custodian, and the coverage. Specify the type/number of drugs included within the database and the thesaurus, taxonomies, or ontologies used for coding drugs and events.*	P2-3
	5b	*Specify the extraction dates and describe and justify all choices used for data pre-processing, including any data transformation or exclusion, if appropriate.*	P3
Variables definition	*6a*	*Describe the study population, including any restriction.*	*NA*
	6b	*Describe the nature and the meaning of key variables assessed in the work.*	P2-3
	6c	*Specify and justify any grouping of drugs or events. For drugs, specify and justify whether active ingredients/trade names/salts were considered and/or the selected role.*	P3
	6d	*Describe any additional data source used, the type of data, and how they interact with ICSRs.*	*NA*
Statistical methods	*7a*	*Present any descriptive analysis performed, specifying variables investigated, statistical tests, and significance thresholds.*	P4
	7b	*Describe the measure(s) selected for the disproportionality analysis including any threshold used to identify signals of disproportionate reporting. Explain the reason for this choice if applicable.*	*P3, Supplementary Table S2*
	7c	*Clearly describe any sensitivity analysis and any tool to control confounding, including any restriction, subgroup,*	P3,4

		stratification, adjustment, or interaction.	
	7d	*Specify the variables and methods used for the case-by-case analysis, including any algorithm or criteria used to assess causality, if performed.*	P3,4
	7e	*Specify any statistical methods used for other data sources.*	*NA*
**Results**			
Participants	*8a*	*Specify the number of individual case safety reports included at each stage, including reasons for exclusion.*	P5, Figure 1
	8b	*Provide key demographic and clinical characteristics of cases, if possible comparing cases with any appropriate reference group.*	*P9*
Disproportionality analysis	*9*	*Present all results including confidence intervals. Present also results of sensitivity analyses, if performed.*	*P4, Table 1*
Case-by-case analysis	*10*	*Present the case-by-case analysis of key variables. Present the causality assessment, if applicable.*	*NA*
**Discussion**			
Key results	*11*	*Discuss key results with reference to study objectives and contextualize them within the current literature and other consulted sources. Clearly discriminate between expected reactions and emerging safety signals.*	*P12-14*
External validity	*12a*	*Discuss the external validity of the results to the general population.*	*P12-14*
	12b	*Discuss the potential relevance of results in clinical practice*	*P15*
	12c	*Propose further study designs if applicable*	*P14, 15*
Limitations	*13*	*Present general limitations, making clear that disproportionality analysis alone cannot prove causation or measure incidence, and specific limitations, including confounding and reporting bias and efforts to mitigate them.*	*P14, 15*
**Declarations**			
	14a	*Provide the source of funding/sponsorship and the role of the funders/sponsors for the present study and for any original study on which the present article is based.*	*P15*
	14b	*Clearly identify potential commercial and intellectual conflicts of interest (e.g., link to any drug/event investigated, whether financial, legal action, or software*	*P15*

		used).	
	14c	*Declare any institutional approval needed or granted in the investigation.*	*P15*
	14d	*Include a statement on data availability, code availability (including the version of the statistical software used), and protocol registration.*	*P15*

Supplementary table 4 Inclusion of the 16 positive renal adverse events associated PTs identified in the combination of ICIs and PPIs in leaflet

PT names	Included in ICIs leaflet	Included in PPIs leaflet
Acute kidney injury	Yes	Yes
Blood creatinine increased	Yes	Yes
Glomerulonephritis	Yes	No
Immune-mediated nephritis	Yes	No
Nephritis allergic	No	No
Nephropathy toxic	No	No
Nephrosclerosis	No	No
Nephrotic syndrome	Yes	No
Proteinuria	Yes	No
Renal impairment	Yes	Yes
Renal tubular acidosis	No	No
Renal tubular atrophy	No	No
Renal tubular disorder	No	No
Renal tubular injury	No	No
Renal tubular necrosis	No	No
Tubulointerstitial nephritis	Yes	Yes
